# Extensive Spread of SARS-CoV-2 Delta Variant among Vaccinated Persons during 7-Day River Cruise, the Netherlands

**DOI:** 10.3201/eid2904.221433

**Published:** 2023-04

**Authors:** Thijs Veenstra, Patrick D. van Schelven, Yvonne M. ten Have, Corien M. Swaan, Willem M. R. van den Akker

**Affiliations:** National Institute for Public Health and the Environment, Bilthoven, the Netherlands (T. Veenstra, Y.M. ten Have, C.M. Swaan, W.M.R. van den Akker);; Municipal Health Services Gelderland-Midden, Arnhem, the Netherlands (P.D. van Schelven)

**Keywords:** COVID-19, severe acute respiratory syndrome coronavirus-2, SARS-CoV-2, coronavirus, viruses, delta variant, coronavirus disease, respiratory infections, ships, river cruise, extensive spread, whole-genome sequencing, disease transmission, infection control, ventilation, government agencies, zoonoses, the Netherlands

## Abstract

We investigated a large outbreak of SARS-CoV-2 infections among passengers and crew members (60 cases in 132 persons) on a cruise ship sailing for 7 days on rivers in the Netherlands. Whole-genome analyses suggested a single or limited number of viral introductions consistent with the epidemiologic course of infections. Although some precautionary measures were taken, no social distancing was exercised, and air circulation and ventilation were suboptimal. The most plausible explanation for introduction of the virus is by persons (crew members and 2 passengers) infected during a previous cruise, in which a case of COVID-19 had occurred. The crew was insufficiently prepared on how to handle the situation, and efforts to contact public health authorities was inadequate. We recommend installing clear handling protocols, direct contacts with public health organizations, training of crew members to recognize outbreaks, and awareness of air quality on river-cruise ships, as is customary for most seafaring cruises.

Cruise ships have long been associated with an increased risk for outbreaks of infectious diseases, illustrated by transmissions of respiratory pathogens and pathogenic microorganisms spreading by the fecal–oral route (*1*–*5*). The special circumstances on a cruise ship with crowded, confined spaces, where fresh air supply is sometimes limited, contributes to the risk for spreading airborne pathogens (*6*–*9*). An additional factor is that the passengers on cruise ships are in general of older age and therefore more susceptible to infections (*10*).

The consequences of an outbreak of an infectious disease on a seafaring cruise can be massive (*11*–*13*). At every port, exchange of passengers occurs, leading to a new risk for introduction of a contagious disease. Besides the financial and commercial consequences, the distance to medical facilities is sometimes considerable, which hinders medical consultation and eventual hospitalization. Therefore, companies organizing seafaring cruises take extensive measures to reduce risks by appointing medically trained personnel, installing care facilities on board, and training personnel to be vigilant about presence of symptomatic passengers that might point to infectious diseases. In addition, prevention plans, outbreak protocols, and procedures for early contact with port health authorities, consistent with provisions of the Maritime Declaration of Health, Annex 8 of the World Health Organization International Health Regulations (*14*), should be installed (*15*,*16*).

In contrast to the preparedness for seafaring cruises, only limited attention is given to those risks on river cruise ships (*17*), although there are many characteristics in common with the larger seafaring cruise ships. In general, river cruises are subject to less regulation concerning medical preparedness (expertise and facilities) because of proximity of shore-based facilities. It has been reported that the number of (river) cruises was increasing worldwide before the pandemic (*18*). Therefore, closer attention is justified.

The outbreak of infection with SARS-CoV-2 on the seafaring cruise ship Diamond Princess in early 2020 gained worldwide attention (*19*), and many studies were directed at conditions on the ship and handling of viral spread among passengers (*20*–*24*). During the COVID-19 pandemic, additional outbreaks on boats and seagoing cruise ships were reported (*12*,*17*,*25*). Sekizuka et al. (*26*) reported a SARS-CoV-2 outbreak at a river-cruise ship sailing the Nile. However, there is only limited awareness of the risk for spread and handling of airborne pathogens on river cruise ships (*27*).

We investigated a large outbreak of infection with SARS-CoV-2 on a river cruise ship sailing in the Netherlands. We focused on virus introduction and spread among passengers and crew members, as well as conditions on the ship that might have contributed to transmission of the virus. Using epidemiologic investigation and genomic sequence analyses, we present a plausible chronicle of spread of the virus among passengers and crew members. We identified some serious issues concerning preparedness of the crew and company. On the basis of this study, we propose several feasible prevention and intervention strategies to mitigate the chance of introduction and (further) transmission of airborne pathogens in a river cruise setting.

## Methods

### Description of Cruise Ship and Demographics of Virus-Positive Passengers

The outbreak of COVID-19 among crew and passengers took place on a 91-m long, 3-deck cruise ship that has capacity for 124 passengers. The ship contains 65 cabins. During the 7-day cruise over rivers in the Netherlands during October 2021, 90% of the cabins were occupied. The exact itinerary and dates are not given because doing so would enable identification of the ship; this anonymity enabled company owners and employees to speak freely to us and to provide valuable insights. We compiled demographics of the persons who tested positive (Table 1). No permission was obtained to contact persons who were not tested or who tested negative for further inquiries.

**Table 1:** Characteristics of passengers and crew who had positive PCR or rapid antigen (self) test results for severe acute respiratory syndrome coronavirus-2 during 7-day river cruise, the Netherlands

Characteristic	Result
All persons	60
Passengers	49 (44% of total passengers)
Crew*	11 (52% of total crew members)
Sex	22 male (37%), 38 female (63%)
Passengers	16 male (33%), 33 female (67%)
Crew	6 male (55%), 5 female (45%)
Age distribution, y	
0–20	2 (3%)
21–40	4 (7%)
41–60	2 (3%)
61–80	33 (54%)
>81	15 (25%)
Missing data	4 (7%)
Symptomatic	52 (87%), 3 missing data (5%)
Passengers	47 (96%)
Crew	6 (55%), 3 missing data (27%)
Vaccinated	56 (93%)
Passengers	49 (100%)
Crew	7 (64%)

*The child of a crew member was included in the crew statistics.

### Epidemiologic Investigation of SARS-CoV-2‒Positive Persons

To investigate and control SARS-CoV-2 infection outbreaks in the Netherlands, the Municipal Health Services (MHS) perform SARS-CoV-2 laboratory testing, collect patient information (date of birth, sex, vaccination status, date of first symptoms, and date of testing) and perform contact tracing. Because participants of the cruise lived throughout the Netherlands, the coordinating MHS sent a questionnaire to all 15 involved MHS locations to collect outbreak-related information from exposed passengers and crew. Anonymized data of persons who had positive results during this outbreak were made available by the MHS (data for virus-negative tested persons were not available), as well as conclusions from the contact-tracing investigation concerning possible transmissions between passengers during the cruise.

### Genetic Characterization and Comparison of Viral Strains

We used nanopore sequencing (Oxford Nanopore Technologies, https://nanoporetech.com) to determine the genomic sequence of the SARS-CoV-2 isolates (*28*). The MHS made a random selection of 20 samples for whole-genome sequencing based on the lowest day of birth in a month. Several of those samples had already been destroyed in the different testing facilities involved, which resulted in the availability of 11 specimens for sequencing. To determine the prevalence and spread of SARS-CoV-2 lineages within the Netherlands, we used the GISAID database (https://www.gisaid.org) and used the Audacity/Instant tool to query for most similar sequences.

### Investigation of Cruise Ship and Circumstances during the Cruise

We collected information about the ship and the conditions during the cruise from the shipping company. Some authors visited the ship and conducted interviews with the owner, the captain, and 3 crew members. A ship sanitation inspector accompanied the interviewers and reviewed the ventilation systems of the ship.

## Results

### SARS-CoV-2 Infections on River Cruise Ship

In October 2021, a local MHS was contacted indirectly by a shipping company, reporting that 2 passengers on their ship had tested positive for SARS-CoV-2 by using rapid antigen self-test. The rapid-antigen tests were available on the ship for crew members and symptomatic passengers. Because the ship was on the 6th day of a 7-day voyage, the municipal health authorities advised reverse transcription PCR testing of all symptomatic persons as soon as possible after disembarking. When the number of identified cases increased strongly after the cruise, all passengers and crew members were encouraged to be voluntarily tested, regardless of symptoms, at the MHS reverse transcription PCR testing facility near their location (free of charge).

One week after the cruise, 60 cases were detected and reported to the MHS, which indicated that at least 49 (44%) of 111 passengers and 11 (52%) of 21 crew members had tested positive for SARS-CoV-2. It is unknown how many passengers and crew members tested negative. All infected passengers and 64% of the crew members were vaccinated (Table 1). This finding raised the question of how SARS-CoV-2 could have been introduced massively onto the ship or alternatively, how the virus could have spread so extensively among passengers and crew members during the cruise; a combination of these 2 factors is also possible.

### Time Line and Epidemiologic Analysis

**Figure 1:**
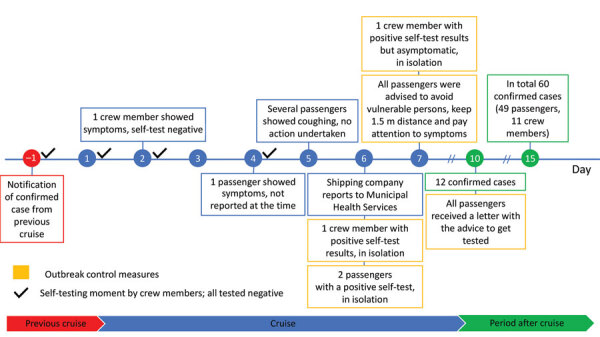
Timeline for COVID-19‒related conditions as part of extensive spread of SARS-CoV-2 Delta variant among vaccinated persons during 7-day river cruise, the Netherlands.

On the basis of information from the municipal health authorities, the crew members, and the questionnaires, we constructed a timeline of the events before, during, and after the river cruise (Figure 1). All crew members, who were the same crew members as on a previous cruise, tested negative for SARS-CoV-2 by using rapid antigen self-tests before departure of the cruise ship. The passengers had to show a document of being vaccinated or proof of a laboratory-confirmed recent infection (or a recent negative test result) and were asked if they had any symptoms before they were allowed to participate in the cruise. Vaccinated at the time consisted of a full series of European Union‒approved vaccines, meaning 2 doses of Pfizer-BioNTech (https://www.pfizer.com), Moderna (https://www.modernatx.com), or AstraZeneca (https://www.astrazeneca.com) vaccines or a single dose of Janssen https://www.janssen.com) vaccine.

At the time of the cruise, no boosters were registered in the Netherlands. Two passengers from the previous cruise stayed on board and were not tested or asked about having symptoms at the start of their second journey. On the day before departure, the company was informed that another passenger from the previous cruise had tested positive for SARS-CoV-2. The 2 passengers and 1 crew member from the previous cruise showed symptoms and tested positive on the 6th day of their second cruise. Although there were facemasks on board, use was not encouraged, even after identification of these positive persons, as was revealed in interviews with passengers.

After the positive cases were identified, the company searched for advice and had difficulties reaching the appropriate authority. Eventually, contact was established with the MHS, which started a contact-tracing investigation. Passengers and members of the crew who had positive results were isolated in their cabins until disembarking. The only 2 passengers who tested positive in a rapid-antigen test on board also shared a cabin. They were isolated together in their cabin until disembarking that night. Crew members who tested positive were isolated individually.

**Figure 2:**
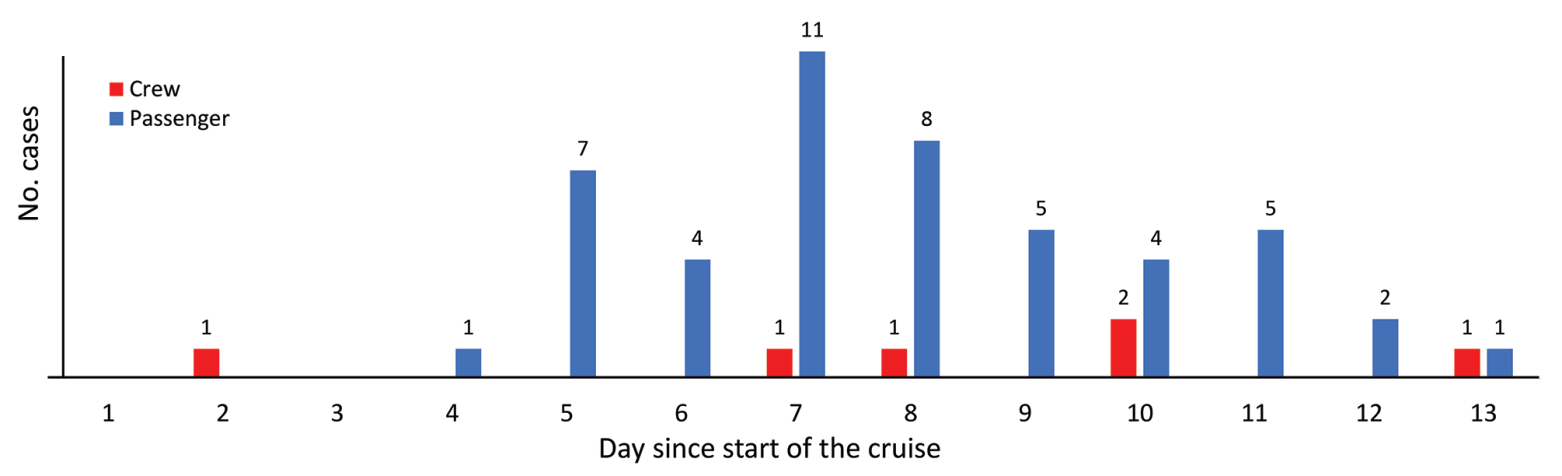
Day of first symptoms for COVID-19‒conditions as part of extensive spread of SARS-CoV-2 Delta variant among vaccinated persons during 7-day river cruise, the Netherlands. Infections were later confirmed by using reverse transcription PCR. The day for first symptoms is unknown for 3 crew members. The cruise ended on day 7.

Because of the infected persons, entertainment on the last evening of the cruise was limited and social distancing between families was encouraged. On the 7th day, a second member of the crew who had a positive test result was isolated. When 12 cases were confirmed 3 days after disembarking, all other passengers and crew members were advised to be tested at the MHS, regardless of symptoms, which resulted in identification of 60 infected persons total. We constructed an epidemiologic curve showing the days of first symptoms for all 60 cases (Figure 2). From the 5th day of the cruise on, larger numbers of persons had symptoms, which reached a peak at the end of the 7-day cruise and gradually decreased in the days thereafter. 

During the contact-tracing investigation, passengers reported to have noticed limited COVID-19‒related measurements. Social distancing was barely practiced, and the use of face masks was not applied. The shipping company confirmed the lack of additional measurements and stated that these measurements were not mandatory at that point during the pandemic.

Retrospectively, a few participants reported that several passengers had serious coughing. During the cruise, several recreational activities took place outside the ship, including a museum visit, an excursion with a tour boat, and a bus trip. There were multiple city walks, combined with visiting restaurants and terraces. Throughout these activities, the passengers primarily remained in groups, and only limited mixing with the public occurred.

### Genomic SARS-CoV-2 Analyses

**Figure 3:**
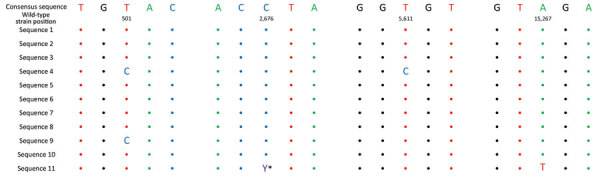
Comparison of whole-genome SARS-CoV-2 sequences obtained from cruise participants during 7-day river cruise, the Netherlands. Only the regions with different nucleotides from the consensus sequence are displayed. Colored circles indicate sequences identity. Sequences classified as the Delta variant of concern were subtyped as AY.126. *Indicates C and T in the same person, possibly co-infection with 2 strains or a mutation that occurred in that person.

We sequenced the SARS-CoV-2 genomes of available isolates from 8 passengers and 3 crew members. All samples were successfully sequenced except for the sample from 1 passenger, for which sequencing of the first 340 nt of the genome was not successful. The 11 strains belonged to the Delta variant of concern (B.1.617.2) and were closely related, with a maximum difference of 2 nt (Figure 3). Of the 723 sequenced isolates in the 4 provinces that were visited during the cruise and the following week, only 2 isolates showed close relatedness (3-nt and 4-nt genomic differences). The SARS-CoV-2 incidence was 24.4 cases/100,000 persons in the Netherlands at that time (https://coronadashboard.rijksoverheid.nl/landelijk/positief-geteste-mensen).

### Use of Ship Facilities during River Cruise

The cruise ship was occupied by 21 crew members and 111 passengers throughout the 7-day river cruise. We provide the sizes of various areas of the ship and their occupancy during the cruise (Table 2). Because the weather was chilly and rainy, the passengers stayed inside, and mostly the interior spaces of the ship were used. The restaurant area was at times crowded because all passengers used the area at the same time for ≈3 hours/day. This location was the only common area where seats were assigned to individual passengers, and this plan had not changed throughout the journey.

**Table 2:** Ventilation systems and maximum occupancy of the various areas of the cruise ship during 7-Day River Cruise

Area	Volume, m^3^ (area × height)	Ventilation system	Maximum occupancy
Cabin	21 (10 × 2.1)	Air supply from corridor and extrusion through the bathroom	2
Corridors	105 (50 × 2.1)	Air from common areas leading to cabins	15
Restaurant	262 (125 × 2.1)	Mechanical recirculation	110
Lounge	440 (200 × 2.2)	Mechanical ventilation, mixing recirculated and fresh air	110
Reception hall	80 (36 × 2.2)	Mechanical ventilation, mixing recirculated and fresh air	50

The seating plan showed no clear clustering of persons who had positive results. Also, no clustering was observed in the layout of the cabins (conclusions from contact-tracing investigation). Available data showed that for 16 cases, both passengers sharing a cabin were infected, whereas for 9 cases, only 1 of the 2 passengers sharing a cabin was infected. Throughout the day, the lounge area, including lounge and bar, was used by small groups of passengers. Passengers in this area were mostly involved in conversations. The crew reported limited crowding during the day, but in the evening the reception area was used extensively for multiple group activities, including a quiz, bingo, and a music performance. Other potential areas of close contact were the narrow corridors, but the crew did not observe crowding there.

The ventilation mechanisms on board established recirculation of air in the restaurant, reception area, and lounge bar (Table 2). In the reception area and lounge bar, the crew had modified the air outlets, mainly situated in the windowsills, to prevent recirculation. The cabins received air from the corridors. The ventilation systems contained elementary filters, but they were not of the quality of high-efficiency particulate air filters (*29*,*30*). In intensively used areas, a fresh air supply was not guaranteed. In the restaurant area, air was only recirculated.

## Discussion

Seafaring cruise ships and river cruise ships have many characteristics in common regarding the use of confined spaces, air quality control, a high occupation level, a similar age group of passengers, duration of voyages, and similar responsibilities of the crew. However, the preparedness for outbreaks of contagious diseases differ considerably between the 2 types of cruises (*27*).

In our study, we investigated an extensive outbreak of SARS-CoV-2 infections among passengers and crew members on a river cruise ship, which affected 60 persons. However, we cannot exclude that some infections might have occurred directly after disembarking. Another limitation of our study is that we do not have insight into the number of persons who tested negative or were not tested at all. The epidemic pattern is consistent with an early introduction of the virus at the cruise and the incubation time of the SARS-CoV-2 Delta variant (peak ≈4–6 days (*31*).

On the basis of combined epidemiologic and genetic analyses, we conclude that the virus was probably introduced to the cruise by persons (crew or the 2 passengers) from the previous cruise. Because genetic information was not available for all persons who had positive results, we cannot exclude that multiple SARS-CoV-2 introductions had taken place. All passengers were fully vaccinated, and this factor probably contributed to mildness of the symptoms but less so to preventing infections (*32*). Most information about the role of the vaccines to protect from disease and reduction of transmissibility of the Delta variant is derived from large-scale surveillance programs and not from specified crowded conditions. The COVID-19 measures taken on the cruise ship did not prevent extensive spread of the virus.

Because pathogens causing respiratory infections are spreading between persons mostly through droplets and aerosols, air flows and air filtering are major parameters, especially when accommodations are cramped and used by many persons simultaneously (*9*). A systematic review of studies on long-distance airborne transmission of SARS-CoV-2 in indoor community settings provides insight into factors contributing to such transmission (*33*); that review identified insufficient air replacement as a critical determinant of transmission. Installation of high-efficiency particulate air filters can be an effective measure to reduce recirculation of airborne pathogens (*34*).

There are no general regulations concerning ventilation in river cruise ships, and regulations for buildings do not apply to those ships in the Netherlands. In this outbreak, the cruise ship had an inferior air circulation and filtering system. Continuous refreshment of the air in all areas on a ship, preferably using fresh outside air, might lower the spread of airborne pathogens. Air recirculation is only an option in combination with proper filtering. The design stage of a cruise ship should take into consideration the risk for spread of microorganisms by air movements within the ship. Later adjustments are in many situations more difficult and sometimes provisional, as was the instance on the ship in this outbreak. The crew should be well aware of the air climate and install clear instructions for refreshing the air in areas of the ship. The COVID-19 pandemic has demonstrated the effectiveness of wearing a face mask in reducing the number of cases (*35*,*36*). As a simple preventive measure, we recommend that face masks be made available for all persons on board so that they can be used without delay when a risk for infection is suspected.

Multiple passengers on the river cruise reported retrospectively that some of the passengers were regularly coughing at the beginning of the cruise. Because it cannot be expected that passengers ask fellow passengers about their health, crew members can be instructed to be attentive to symptoms of passengers that might indicate an infectious disease and address the situation.

A major problem is that not all symptoms, such as coughing, general malaise, and fever, can be exclusively ascribed to an infectious cause and that the decision to take additional action is a difficult one. The crew can be trained to recognize potential symptoms of infectious diseases and handle these symptoms accordingly. A solution can be that in instances of doubt, the crew seek advice from an appropriate health agency. For the cruise we describe, crew members made contact with public health authorities indirectly, even using private telephone numbers of health officials. Prearranged points of contact provided by health authorities would increase efficiency and effectivity in an outbreak situation. Thus, the combination of well-instructed crew members and the initiative to handle a suspicious situation, including contacting health officials, is a crucial issue.

This outbreak shows the need for preventive measures and vigilance for infectious diseases in river cruise operations. Although full prevention of the spread of infectious agents during a cruise is hard to achieve because passengers can embark during their incubation period and (subclinical) carriership, several simple and effective measures can be taken. Compared with those for seagoing cruises, only minimal requirements for the prevention of spread of infectious diseases on river cruises are available (*37*). Standardized preventive measures would support companies in reducing risk for infectious disease outbreaks, and vigilance on board and the involvement of health authorities could support early detection and response in case of an outbreak. Awareness, training of staff, presence of face masks, and an infrastructure for direct contact with health authorities should be part of a general standard for river cruise operations.
